# Does using 3D printed models for pre-operative planning improve surgical outcomes of foot and ankle fracture fixation? A systematic review and meta-analysis

**DOI:** 10.1007/s00068-022-02176-7

**Published:** 2022-11-24

**Authors:** Lea Wood, Zubair Ahmed

**Affiliations:** 1 College of Medical and Dental Sciences, University of Birmingham, Edgbaston, Birmingham, B15 2TT UK; 2 Neuroscience and Ophthalmology, Institute of Inflammation and Ageing, College of Medical and Dental Science, University of Birmingham, Edgbaston, Birmingham, B15 2TT UK; 3 Centre for Trauma Sciences Research, University of Birmingham, Edgbaston, Birmingham, B15 2TT UK

**Keywords:** 3D printing, 3D models, Foot, Ankle, Pre-operative planning, Fracture

## Abstract

**Purpose:**

The systematic review aims to establish the value of using 3D printing-assisted pre-operative planning, compared to conventional planning, for the operative management of foot and ankle fractures.

**Methods:**

The systematic review was performed according to PRISMA guidelines. Two authors performed searches on three electronic databases. Studies were included if they conformed to pre-established eligibility criteria. Primary outcome measures included intraoperative blood loss, operation duration, and fluoroscopy time. The American orthopaedic foot and ankle score (AOFAS) was used as a secondary outcome. Quality assessment was completed using the Cochrane RoB2 form and a meta-analysis was performed to assess heterogeneity.

**Results:**

Five studies met the inclusion and exclusion criteria and were eventually included in the review. A meta-analysis established that using 3D printed models for pre-operative planning resulted in a significant reduction in operation duration (mean difference [MD] = − 23.52 min, 95% CI [− 39.31, − 7.74], *p* = 0.003), intraoperative blood loss (MD = − 30.59 mL, 95% CI [− 46.31, − 14.87], *p* = 0.0001), and number of times fluoroscopy was used (MD = − 3.20 times, 95% CI [− 4.69, − 1.72], *p* < 0.0001). Using 3D printed models also significantly increased AOFAS score results (MD = 2.24, 95% CI [0.69, 3.78], *p* = 0.005), demonstrating improved ankle health.

**Conclusion:**

The systematic review provides promising evidence that 3D printing-assisted surgery significantly improves treatment for foot and ankle fractures in terms of operation duration, intraoperative blood loss, number of times fluoroscopy was used intraoperatively, and improved overall ankle health as measured by the AOFAS score.

## Introduction

Foot and ankle fractures are common lower extremity injuries, with ankle fractures comprising 17% of all fractures requiring hospitalisation [1]. The incidence of foot fractures has been reported as 142.3/100,000 person-years and the overall incidence of foot and ankle fractures combined has been calculated to be 25.87/10,000 person-years [2, 3]. In the tarsal region, calcaneal fractures predominate, accounting for approximately 65% of tarsal injuries [4]. Often caused by falls from height in working-aged males, their economic impact resulting from disability is disproportionate to their incidence [5]. In 2016/17, the cost of inpatient hospital care for ankle fractures in England was estimated to be over £63 million [6]. Fractures in the foot and ankle region, therefore, pose a significant burden on patients and healthcare systems. These issues are compounded by the complex anatomy of the foot and ankle, the high number of articular surfaces, and their function as weight-bearing structures [7, 8]. For these reasons, early surgical fixation is recommended for unstable fractures of the ankle [9]. Conventionally, these surgeries are planned using 2-dimensional (2D) imaging modalities, such as computed tomography (CT) and X-ray imaging [10]. Such 2D renderings, however, fail to accurately depict the structural complexities of bony fractures, which may lead to imperfect reconstruction. This is problematic in the context of foot and ankle fractures, as improper reduction can lead to post-traumatic osteoarthritis of articular surfaces, which in turn can greatly impair mobility [4, 10].

3D printing, also known as additive manufacturing or rapid prototyping, is a rapidly expanding technology that is beginning to revolutionise the medical and healthcare industry [11, 12]. Since its inception in the 1980s for use in design and engineering, it has more recently been introduced in a medical context and is now quickly gaining momentum globally [12–15]. Initially introduced in maxillofacial surgery, the technology can now be applied in various surgical specialties [12, 15]. Within these, 3D printing technology is used for a variety of applications including pre-operative planning, patient and medical student education, and the manufacture of surgical implants and prostheses [12, 16]. It has proven itself particularly useful for inexperienced surgeons by allowing them to familiarise themselves with surgical techniques prior to implementation on patients [17, 18]. In this context, the 3D printed models facilitate surgery by enabling an enhanced understanding of the anatomy, improved communication between clinicians and patients, and improved tailoring of both tools and surgical techniques to the individual fracture [19]; as such, this new approach constitutes a form of personalised medicine, which is central to contemporary healthcare.

Briefly, the 3D printing process consists of three stages: image acquisition, image processing and 3D printing. The first step involves using imaging modalities such as CT images to obtain an image of the fracture site. These are then stored in a digital imaging and communications in medicine (DICOM) format. Then, image processing entails the conversion of the DICOM images to standard triangulation language (STL) files using software packages, whereby the images are segmented to create a triangular mesh. From these, the 3D model is printed [16, 19]. Numerous 3D printing technologies exist, including stereolithography, binder jetting, material jetting, material extrusion, and powder-bed fusion [11].

Increasing interest in 3D printing-assisted surgery has been accompanied by mounting evidence pointing towards its efficacy in pre-operative planning, with promising results being presented in the research of various musculoskeletal injuries [20–22], though much of this research has focussed on acetabular and tibial plateau fractures [23–27]. Existing reviews have investigated 3D printing-assisted surgery in calcaneal and pilon fractures in isolation [28, 29], however, the aim of this systematic review was to determine the efficacy of 3D printing in the pre-operative planning of combined foot and ankle fractures. The results of our study demonstrate that 3D printing-assisted surgery significantly improves treatment for foot and ankle fractures in terms of operation duration, intraoperative blood loss, number of times fluoroscopy was used intraoperatively, and improved overall ankle health as measured by the American Orthopaedic Foot and Ankle Score (AOFAS).

## Methods

### Literature search

The review was completed according to the Preferred Reporting Items for Systematic Reviews and Meta-Analyses (PRISMA) guidelines [30]. On 22nd June 2022, three databases were searched by two independent reviewers (L.W. and Z.A.): EMBASE, MEDLINE and Web of Science. The search strategy involved variations of the words: ‘three-dimensional printing’, ‘additive manufacturing’, ‘rapid prototyping’, ‘bone fractures’, ‘foot’, ‘ankle’ ‘calcaneus’, ‘tarsal’, ‘cuneiform’, ‘cuboid’, ‘talus’, ‘pilon’, ‘navicular’, ‘malleolus’, ‘metatarsal’, ‘phalanx’, sesamoid’ and ‘talocrural’, combined with Boolean operators. All searches were restricted to English language only and a publication date of 2012 or later. Duplicates were removed, then titles and abstracts of the search results were screened against the eligibility criteria. Subsequently, the full texts of selected studies were assessed against the eligibility criteria. A grey literature search was completed using Ethos, ClinicalTrials.gov and the Cochrane Library.

### Inclusion and exclusion criteria

Inclusion criteria were as follows: (i) randomised controlled trials (RCTs), (ii) studies comparing 3D printing-assisted surgery to conventional surgery, (iii) treatment of fractures of the foot and/or ankle, (iv) published in 2012 or later, (v) outcome measures include at least 2 of: intraoperative blood loss, operation duration and fluoroscopy time. The following exclusion criteria were applied: (i) any other study type, (ii) non-English language, (iii) non-human studies, (iv) cadaveric studies, (v) studies investigating primarily patients with pathologies other than trauma (e.g. tumours, osteoporosis), (vi) studies published before 2012.

### Data collection

Data were collected from the included studies using data collection forms. The forms were created and piloted against two studies, then amended to suit the review. The data collected included study characteristics, participant characteristics, and outcome data. After piloting, data collection was carried out by both authors independently and disagreements were resolved with discussions. Data were collected prior to quality assessment to limit reporting bias. Data were transferred from the data collection forms into tables of study characteristics and outcomes.

### Quality assessment

The risk of bias (RoB) was assessed independently by the two reviewers (L.W. and Z.A.), with discussion to resolve disagreements. The Cochrane Risk of Bias 2 (ROB2) tool was used, given that all included studies were RCTs [31]. Five domains were assessed: bias arising from the randomisation process, bias due to deviations from the intended interventions, missing outcome data, bias in the measurement of the outcome, and bias in the selection of reported results. Forms were completed for each outcome of each study, and then an overall quality assessment was determined for each study.

### Data synthesis and statistical analysis

Review Manager (RevMan 5.4, Cochrane Informatics & Technology, London, UK) was used to determine the *Q* and *I*^2^ statistics (in percentages) to establish variation between the studies attributed to heterogeneity. An *I*^2^ value greater than 50% was considered significant heterogeneity. A meta-analysis of a subgroup of studies that reported quantitative data for the outcomes: operation duration, intraoperative blood loss, number of times fluoroscopy was used, and American Orthopaedic Foot and Ankle Society (AOFAS) Ankle-Hindfoot Rating System scores in control and 3D printing intervention groups were conducted in RevMan 5.3 (Cochrane Informatics & Technology), using the dichotomous data function, and employing a random effects model.

## Results

### Study selection

**Fig. 1:**
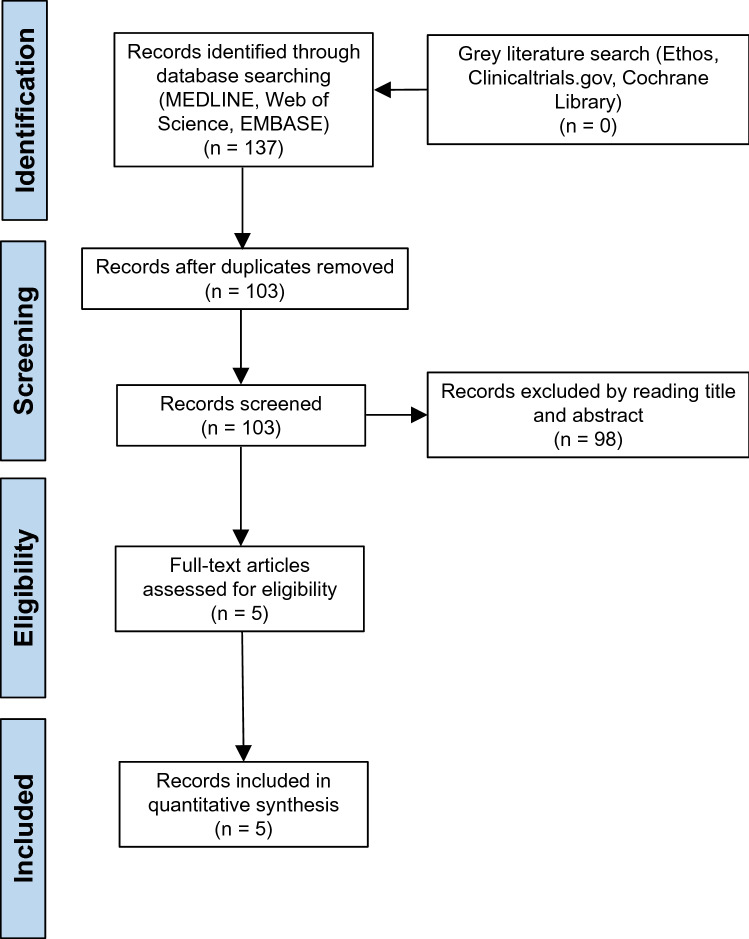
PRISMA flow chart of included studies

After conducting the systematic search of the information following the PRISMA strategy, 137 studies were found in MEDLINE, Web of Science and EMBASE (Fig. 1). From these, 103 studies remained after de-duplication. After screening titles and abstracts, 98 studies were excluded because they did not suit the eligibility criteria. Therefore, five studies were eligible for full text analysis and were hence included in the final analysis. The grey literature search completed using Ethos, ClinicalTrials.gov and the Cochrane Library yielded no further relevant studies beyond those already selected from the initial search.

### Study characteristics

In the five studies included in this review [32–36], a total of 316 patients were analysed, 153 of which being allocated to the 3D printing group and 163 being allocated to the control group. Sample sizes ranged from 15 to 45 in the 3D group and 15 to 48 in the control group (Table 1). All studies were small-scale RCTs, with an overall mean sample size of 63.2 patients. In all studies, the intervention group involved the use of 3D printed models for pre-operative planning and simulation of the surgery. These were compared to a control group in which no 3D printed model was used. Three of the five studies investigated calcaneal fractures [33, 35, 36]; in these studies, the severity of calcaneal fracture included varied, with Dai et al. including only Sander’s types II and III [33], and the remaining two studies including types II to IV [35, 36]. Besides calcaneal fractures, trimalleolar and pilon fractures were also investigated [32, 34]. All included studies except that by Yang et al. provided inclusion and exclusion criteria. Four of the five studies used Mimics software [32–35], with Ozturk et al. [36] using Autodesk Meshmixer. The 3D ORTHO Waston Med Inc. 3D printer was used most often [33–35], though a FlashForge printer was used by Yang et al. [32]. Ozturk et al. [36] failed to disclose information on which printer was used. Besides the outcomes analysed in this meta-analysis (operation duration, intraoperative blood loss, number of times fluoroscopy was used, and AOFAS score), other outcomes assessed in the studies include Böhler and Gissane angles, calcaneal width and height, VAS scores, Burwell and Charnley scores, fracture union times, complication rates, and questionnaires. Four of the included studies were produced in China [32–35], and one originated from Turkey [36].

**Table 1:** Characteristics of included studies

Study (first author, year, country)	Fracture location and type	Inclusion and exclusion criteria	3D software and printer	Treatment type		Sample size (n)		Mean age (years)		Sex (M, F)		Mean follow-up (months)		Outcomes
				3D	Control	3D	Control	3D	Control	3D	Control	3D	Control	
Yang, 2016, China	Trimalle-olar Lauge-Hansen type IV supination-external rotation and type IV pronation-external rotation	n/s	Mimics 10.01, FlashForge Ltd	Surgery with 3D printed models used for pre-operative planning and simulation	Surgery with 3D reconstruction image used for pre-operative planning	15	15	36.5		16, 14		n/s	n/s	Questionnaire, operation duration, intraoperative blood loss
Zheng, 2017, China	Calcaneal Sanders types II, III and IV	I: fresh closed fracture (within 1 week of injury), unilateral calcaneal fracture, contralateral normal calcaneus should not have any fracture/ deformity/ history of surgery, min. 12 months follow-up E: contralateral calcaneus fractures and/or dislocations, Sanders type I calcaneus fractures, pathological fractures, open fractures, severe soft tissue injury (Tscherne and Oestern closed grade II and grade III), multiple fractures	Mimics 17.0, 3D ORTHO Waston Med Inc	Bone graft, plate and screws with 3D printed models used for pre-operative planning and simulation, selection of orthopaedic hardware and intraoperatively to guide surgery	Bone graft, plate and screws with measurements used to select orthopaedic hardware	35	40	44.5 ± 8.0	46.7 ± 6.2	19, 16	25, 15	14.9 ± 1.9	14.7 ± 2.0	Operation duration, intraoperative blood loss, fluoroscopy time, fracture union time, Böhler angle, Gissane angle, calcaneal width, calcaneal height, VAS score, AOFAS score, complications, questionnaire
Zheng, 2018, China	Pilon AO/OTA types 43-C1, 43-C2 and 43-C3	I: age > 18, fresh closed fractures (within 2 weeks from injury), unilateral pilon fracture, the contralateral normal tibia should not have any fracture/ deformity/ history of surgery, min. 12 months follow-up E: contralateral tibia fractures and/ or dislocations, old/ pathological fractures, severe soft tissue injury (AO closed soft tissue injury grades IC4 and IC5), multiple fractures	Mimics 17.0, 3D ORTHO Waston Med Inc	Plate and screws with 3D printed models used for pre-operative planning and simulation, selection of orthopaedic hardware and for reference intraoperatively	Plate and screws with measurement used to select orthopaedic hardware	45	48	41.2 ± 9.3	42.5 ± 9.0	35, 10	31, 17	20.5 ± 3.7	19.9 ± 3.3	Operation duration, intraoperative blood loss, fluoroscopy time, fracture union time, Burwell and Charnley score, AOFAS score, VAS score, questionnaire
Ozturk, 2020, Turkey	Calcaneal Sanders types IIA, IIB, IIIAB, IIIAC, IIIBC and IV	I: fresh, closed, unilateral calcaneal fracture, normal, non-fractured contralateral calcaneal anatomy, complete CT images, no associated injuries E: bilateral calcaneal fracture, previous ankle/ foot and calcaneal surgery, pathological fractures, open fractures, existing ankle and foot deformity, severe soft tissue injury, associated injuries	Autodesk Meshmixer 2017, n/s	Plate and screws with 3D printed models used for pre-operative planning and simulation and selection of orthopaedic hardware	Plate and screws with measurements used to select orthopaedic hardware	18	19	45.2 ± 9.1	41.1 ± 12.3	16, 2	16, 3	15.4 ± 6.8	15.5 ± 7.0	Operation duration, instrumentation time, intraoperative blood loss, fluoroscopy time, Böhler angle, Gissane angle, calcaneal width, calcaneal facet height, AOFAS score, fracture union time, questionnaire
Dai, 2021, China	Calcaneal Sanders types II and III	I: DIACF (> 2 mm) including Sanders types II and III, unilateral fracture, closed fracture, fresh fracture (within 1 week from injury), age ≥ 18 years, min. 12 months follow-up E: Sanders type IV, bilateral and open fractures, pathological fractures, medical contraindications, refused to accept treatment plan	Mimics 17.0, 3D ORTHO Waston Med Inc	Calcium sulfate cement and hollow screws with 3D printed models used for pre-operative planning and simulation and intraoperatively to guide surgery	Calcium sulfate cement and hollow screws with measurements used to select orthopaedic hardware	40	41	44.1 ± 12.9	46.1 ± 12.6	32, 8	30, 11	28.3 ± 7.7	31.0 ± 7.2	operation duration, intraoperative blood loss, fluoroscopy times, Böhler angle, Gissane angle, calcaneal width, calcaneal height, AOFAS score, complications, questionnaire

*I* inclusion criteria, *E* exclusion criteria, *n/s* not stated, *M* male, *F* female, *AOFAS* American Orthopaedic Foot and Ankle Score, *VAS* Visual Analogue Scale, *AO/OTA* AO Foundation/Orthopaedic Trauma Association

### Patient characteristics

Overall, the mean age of patients included in the review was 42.46 years (Table 1). All studies reported a high male-to-female ratio, with overall 69.6% of all study participants being male. The mean time to follow-up values were similar between groups in all studies which provided this information; the shortest follow-up times were reported by Zheng et al. [35] in 2017 (3D: 14.9 ± 1.9 months, control: 17.7 ± 2.0 months) and the longest follow-up times were reported by Dai et al. [33] (3D: 28.3 ± 7.7 months, control: 31.0 ± 7.2 months).

### Quality assessment

**Fig. 2:**
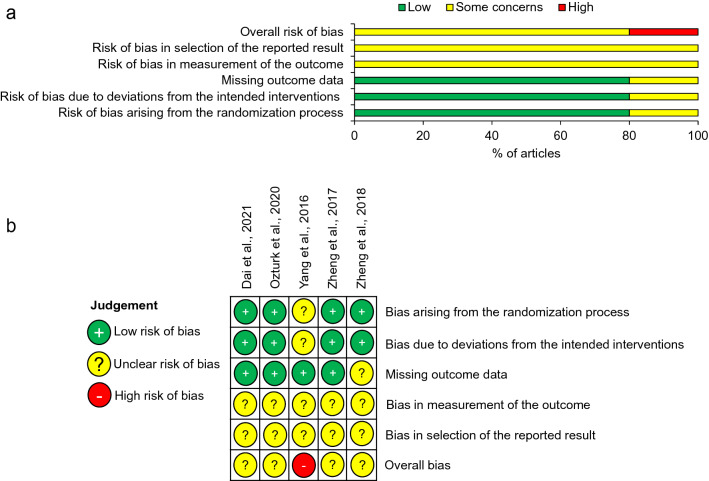
RoB Assessments. **a** Summary of RoB assessments for individual studies included in the systematic review, **b** Summary RoB assessments according to RoB2 bias domains

The overall risk of bias was classified as ‘some concerns’ in four of the five studies and ‘high risk’ in one study (Fig. 2). The study by Yang et al. [32] was categorised as high risk because four of the five domains received an evaluation of ‘some concerns’. The domains: ‘selection of the reported results’ and ‘measurement of the outcome’ received a rating of ‘some concerns’ in all studies. The former of these was due to a lack of analysis intentions being available for any of the included studies. The latter was due to the nature of AOFAS scoring; since the intervention is surgical, it could not be blinded and AOFAS scoring relies on a combination of patient-reported and clinician-reported answers.

## Results of individual studies

Yang et al. (2016) recorded an operation duration of 71 ± 23 min and 98 ± 20 min in the 3D and control groups respectively (*p* =  < 0.05) (Table 2). The intraoperative blood loss recorded in the 3D group was significantly lower than that in the control group (3D: 65 ± 26 mL, control: 90 ± 38 mL, *p* < 0.05). Neither the number of times fluoroscopy was used nor AOFAS score were reported in this study [32].

**Table 2:** Primary and Secondary Outcomes from Individual Studies

Study (first author, year)	Primary outcomes						Secondary outcome	
	Operation duration (mins)		Intraoperative blood loss (mL)		Fluoroscopy use (times used, *n*)		AOFAS score	
	3D	Control	3D	Control	3D	Control	3D	Control
Yang, 2016	71 ± 23	98 ± 20	65 ± 26	90 ± 38	n/s	n/s	n/s	n/s
Zheng, 2017	71.4 ± 6.8	91.3 ± 11.2	226.1 ± 22.6	288.7 ± 34.8	5.6 ± 1.9	8.6 ± 2.7	87.6 ± 7.6	85.8 ± 9.0
Zheng, 2018	74.1 ± 8.2	90.2 ± 10.9	117.1 ± 20.7	159.8 ± 26.5	7.6 ± 2.2	11.0 ± 2.9	87.4 ± 8.7	84.7 ± 9.0
Ozturk, 2020	83.3 ± 4.6	130.0 ± 5.8	83.6 ± 4.6	105.1 ± 5.6	6.8 ± 1.4	11.7 ± 1.5	86.1 ± 4.1	84.5 ± 4.9
Dai, 2021	46.7 ± 7.2	55.1 ± 8.8	14.3 ± 5.7	18.7 ± 6.0	9.5 ± 1.8	11.2 ± 1.8	90.4 ± 5.3	87.7 ± 6.4

Primary outcomes include operation duration, intraoperative blood loss and fluoroscopy use. AOFAS score was used as a secondary outcome. *n/s* not stated

In 2017, Zheng et al. [35] reported a significantly reduced operation duration (3D: 71.4 ± 6.8 min, control: 91.3 ± 11.2 min, *p* < 0.0001), intraoperative blood loss (3D: 226.1 ± 22.6 mL, control: 288.7 ± 34.8 mL, *p* < 0.0001) and number of times fluoroscopy was used (3D: 5.6 ± 1.9 times, control: 8.6 ± 2.7 times, p < 0.0001) in the 3D group compared to the control. The AOFAS score was marginally higher in the 3D group (3D: 87.6 ± 7.6, control: 85.8 ± 9.0), although this was not statistically significant (*p* = 0.341).

In 2018, Zheng et al. [34] reported that using 3D printing for pre-operative planning was successful in reducing operation time (3D: 74.1 ± 8.2 min, control: 90.2 ± 10.9 min, *p* < 0.001), blood loss volume (3D: 117.1 ± 20.7 mL, control: 159.8 ± 26.5 mL, *p* < 0.001) and number of times fluoroscopy was used (3D: 7.6 ± 2.2 times, control: 11.0 ± 2.9 times, *p* < 0.001). Again, the AOFAS score was increased with the use of 3D printing (3D: 87.4 ± 8.7, control: 84.7 ± 9.0) but this change was not significant (*p* = 0.149).

A similar outcome was reported by Ozturk et al. [36]; the operation duration (3D: 83.3 ± 4.6 min, control: 130.0 ± 5.8 min, *p* < 0.0001), intraoperative blood loss (3D: 83.6 ± 4.6 mL, control: 105.1 ± 5.6 mL, *p* < 0.0001) and number of times fluoroscopy was used (3D: 6.8 ± 1.4 times, control: 11.7 ± 1.5 times, *p* < 0.0001) were all significantly lower in the 3D group. The AOFAS score was higher in the 3D group, though again not significantly (3D: 86.1 ± 4.1, control: 84.5 ± 4.9, *p* = 0.278).

Dai et al. [33] reported significantly reduced operation duration (3D: 46.7 ± 7.2 min, control: 55.1 ± 8.8 min, *p* < 0.001), intraoperative blood loss (3D: 14.3 ± 5.7 mL, control: 18.7 ± 6.0 mL, *p* < 0.001) and number of times fluoroscopy was used (3D: 9.5 ± 1.8 times, control: 11.2 ± 1.8 times, *p* < 0.001) with the use of 3D models, and a significant increase in AOFAS score (3D: 90.4 ± 5.3, control: 87.7 ± 6.4, *p* = 0.039).

**Fig. 3:**
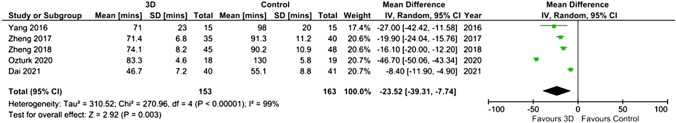
Meta-analysis of operation duration in control versus 3D printed subjects

Meta-analysis of the studies demonstrated that operation duration (Fig. 3) was significantly reduced when 3D printed models were used, with a mean difference (MD) of − 23.52 min (95% CI [− 39.31, − 7.74], *p* = 0.003, *I*^2^ = 99%) [32–36].

**Fig. 4:**
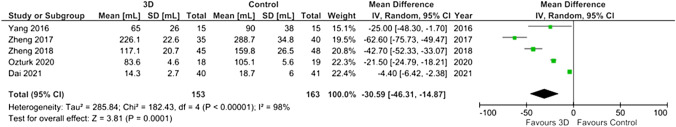
Meta-analysis of intraoperative blood loss during the operation in control versus 3D printed subjects

Intraoperative blood loss (Fig. 4) was also significantly reduced (by MD = 30.59 min) in the intervention group compared to the control in all studies [32–36] (95% CI [46.31, − 14.87], *p* = 0.0001, *I*^2^ = 98%).

**Fig. 5:**
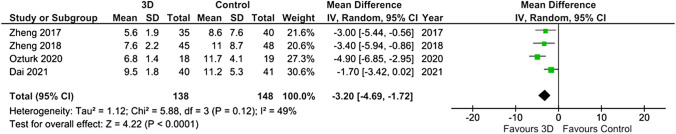
Meta-analysis of the number of times fluoroscopy was used in control versus 3D printed subjects

A meta-analysis of the number of times fluoroscopy was used during surgery (Fig. 5) did not include data from Yang et al. [32], as this data was not reported in the study. Thus, this analysis was carried out on a subgroup of 4 studies [33–36]. Again, all studies reported a significant decrease in number of times fluoroscopy was used (MD = − 3.20 min, 95% CI [− 4.69, − 1.72], *p* < 0.0001, *I*^2^ = 49%).

**Fig. 6:**
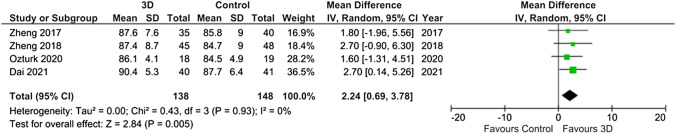
Meta-analysis of AOFAS scores in control versus 3D printed subjects

Finally, a meta-analysis of AOFAS score (Fig. 6) also excluded the study by Yang et al. [32] due to a lack of data. Therefore, 4 studies were included [33–36]. The meta-analysis reported a statistically significant increase in AOFAS score in the intervention group compared to the control group (MD = 2.24, 95% CI [0.69, 3.78], *p* = 0.005, *I*^2^ = 0%).

## Discussion

The objective of this systematic review was to determine whether using 3D printed models for pre-operative planning improved the surgical outcomes of foot and ankle fracture fixation. Only five studies met our inclusion/exclusion criteria and were all RCTs assessing 3D printing versus no 3D printing assistance in foot and ankle fracture fixation. We show that use of 3D printing is promising in planning foot and ankle fracture fixation as it shortens the operation duration, reduces intraoperative blood loss and leads to improved AOFAS outcome scores. To our knowledge, this is the first study to assess 3D printing in foot and ankle fracture fixation, with our results supporting its use for improved outcomes.

Foot and ankle fractures are common injuries, with an estimated incidence of approximately 25/10,000 person-years [3]. Encompassing various small bones and articular surfaces, this region is anatomically complex, and thus generates challenging fractures when afflicted with high-energy trauma [10, 37]. It follows that reconstruction is often carried out operatively, using 2D images for pre-operative planning [8, 9]. Unfortunately, 2D images often fail to accurately capture the complexities of fractures, resulting in complicated surgeries with a high chance of intraoperative difficulties including substantial blood loss and intraoperative fluoroscopy use [38].

The findings presented in this systematic review are yet to be clinically validated but shortened operation durations are unlikely to be clinically relevant but may reduce operating theatre costs and perhaps blood loss. However, blood loss occurring during an average of 23 min shorter duration, might not be clinically relevant since the loss blood in that time is unlikely to lead toa state where blood transfusion is required. The American Association of Blood Banks and NICE guidelines recommend adhering to a restrictive transfusion strategy (7 to 8 g/dL) in hospitalised, stable patients [17]. Shorter duration of the operation may pose some benefits for both the patient and healthcare provider and are especially pertinent to hospitals with limited resources and whose operating theatres are in high demand. Our finding that the intraoperative use of fluoroscopy was reduced using 3D printed models may be clinically relevant; fluoroscopy is essential in guiding surgery, as it enables the surgeon to visualise orthopaedic hardware placement and can guide decision-making. However, a recent study with a sample size of 100 patients found no difference in the number of revision surgeries, complications, foot and ankle outcome score (FAOS), AFOAS or Short-Form 36 (SF-36) scores nor incidence of post-traumatic osteoarthritis at the 2 years follow-up time, between 2 and 3D fluoroscopy [39]. Using 3D models for pre-operative surgical planning, however, the need for fluoroscopy can be reduced because the surgeon has an enhanced understanding of the fracture anatomy before the surgery even begins; simulation of the surgery on the 3D model further allows the surgeon to make detailed decisions such as screw placement without needing to do so intraoperatively. However, these patients should also be evaluated at the 2 year follow-up stage to establish if these early benefits of 3D printed modelling are sustained.

Fewer intraoperative uses of fluoroscopy result in greater safety from radiation exposure for both patients and clinicians [11]. The AOFAS score encompasses measures of pain, function, and alignment of the fracture site; higher scores imply a more successful surgical fixation. It is likely that higher AOFAS scoring was attained in the intervention group because using 3D printed models in pre-operative planning and simulation allows surgeons to become more familiar with the anatomy and prepared for complications, therefore enhancing their ability to precisely fix the fracture. The results of the present review are consistent with existing literature; when investigating its use in acetabular fractures, a recent systematic review identified that 3D printing for pre-operative planning significantly improved surgery in terms of surgical time, blood loss, quality of reduction and clinical outcomes [23]. These results are echoed in further reviews exploring various fracture types [26, 28, 29, 39].

A key strength of the review was the inclusion of exclusively RCTs; this study type is the gold standard for interventional studies due to its rigour and high level of evidence. Hence, the credibility of the review is enhanced with the use of RCTs. However, whilst a highly statistically significant improvement was identified for all outcomes in this review, the results of the operation duration and intraoperative blood loss meta-analyses must be interpreted with caution due to the high degree of heterogeneity of the included studies, which may negatively affect the reliability of the presented results. The high level of heterogeneity in these outcomes is likely due to the review including different fracture types in its assessment. Additionally, Yang et al. [32] provided little information about their methodology. It is therefore possible that their methodology varied compared to those of the other researchers, and thus introduced methodological heterogeneity. This may explain why the meta-analyses of the number of times fluoroscopy was used and AOFAS score generated considerably lower *I*^2^ values, as the study by Yang et al. was excluded from these analyses since these outcomes were not reported in the study [32]. Caution must also be taken because two of the studies were completed by the same primary author. As the review includes an already small number of studies, there is a risk that mistakes by this one author could disproportionately affect the results of the systematic review. However, because the studies were not produced by an identical research team, and because neither of these studies were categorised as having a high risk of bias in the quality assessment, this is unlikely. Should research progress in this field, a greater body of literature may facilitate future systematic reviews which include a greater number of high-quality RCTs from a broader range of institutions.

## Limitations

Owing to time constraints and limited resources, the review was conducted using English language literature only. This presents a language bias and restricts the body of evidence that can be included. This is especially relevant to research on the use of 3D printing in the medical setting, as an abundance of existing literature is non-English [28]. Thus, in future, it would be beneficial to complete further systematic reviews which include literature from a greater pool of areas and languages. Moreover, the quality of a systematic review relies heavily on the quality of the individual studies included. Since these all presented a medium to high risk of bias, the quality of the review is restricted. This is compounded by the fact that all studies in the review were completed in Asia and four of the five studies originated from one country: China. This may limit the review’s relevance to Western medical practices. In future, research from a larger group of institutions is warranted to reflect the global use of 3D printing technology. Given the growing prevalence of 3D printing technology in Western hospitals, this appears attainable in the near future [12, 15].

Whilst a reduction in blood loss and operation duration definitively demonstrates beneficial effects of using 3D printing technology for pre-operative planning, no information was available for the time taken to prepare the models in the included studies. Without data on lead times, the overall time taken for the complete intervention process cannot be compared. Therefore, it is not possible to deduce whether or not the time taken to produce models and the cost of synthesis would negate the reduced cost of shorter operations. Additional information about the time taken to print the models and complete surgical simulation would likely provide better context regarding the efficiency of this method compared to conventional surgery; future efforts should therefore attempt to investigate the financial and practical benefits of using 3D printing-assisted surgery. Further research on lead times would be especially relevant in an emergency trauma context, since these wounds require urgent care [40]. Given that it can take several hours to produce a 3D model [15], it is unlikely that current technology would permit the use of 3D models in these cases. Nevertheless, more modern 3D printing technologies such as CLIP enable much faster ad hoc printing, which could introduce new opportunities for urgent trauma care [12]. Finally, when considering the efficacy of a surgical intervention, clinical outcomes are equally as important as surgical outcomes. However, because this review focussed on surgical outcomes, these factors were beyond the scope of the review; future research should more thoroughly investigate clinical parameters such as complication rate and fracture union time. This will allow researchers to ensure 3D printing technology can reliably improve the standard of care given to patients.

## Conclusion

The present review presents promising evidence to suggest a surgical benefit to using 3D printed models in the pre-operative planning of foot and ankle fracture fixation; using 3D printed models in pre-operative planning significantly reduced operation duration, intraoperative blood loss and number of times fluoroscopy was used, and significantly increased AOFAS scores. In future, research originating from a greater pool of countries may be useful in substantiating these results. Further research on the use of 3D printing in this context should focus not only on surgical outcomes but should expand to cover clinical outcomes in greater detail, as well as investigating financial (cost of printing, reduction in cost of surgical theatre) and practical (software training, printing time) outcomes in greater detail.

## Data Availability

All data is available within this manuscript.
